# First insulinization with basal insulin in patients with Type 2 diabetes in a real-world setting in Asia

**DOI:** 10.1111/j.1753-0407.2011.00137.x

**Published:** 2011-09

**Authors:** Shih-Tzer TSAI, Faruque PATHAN, Linong JI, Vincent Tok Fai YEUNG, Manoj CHADHA, Ketut SUASTIKA, Hyun Shik SON, Kevin Eng Kiat TAN, Yupin BENJASURATWONG, Thy Khue NGUYEN, Farrukh IQBAL

**Affiliations:** 1 Taipei Veterans General HospitalTaipei, Taiwan; 2 Department of Clinical EndocrinologyBirdem, Dhaka, Bangladesh; 3 Department of Endocrinology, Peking University People's HospitalBeijing, China; 4 Centre for Diabetes Education and Management, Our Lady of Maryknoll HospitalHong Kong; 5 PD Hinduja National Hospital and Medical Research CentreMumbai, India; 6 Department of Internal Medicine, Faculty of Medicine, Udayana University/RSUP DenpasarDenpasar, Indonesia; 7 Department of Internal Medicine, The Catholic University of Korea, Uijeongbu St Mary's HospitalGyeonggi-do, Korea; 8 Mount Elizabeth Medical CentreSingapore; 9 Endocrine Unit, Department of Medicine, Phramongkutklao HospitalBangkok, Thailand; 10 Department of Endocrinology, Medical University of Ho Chi Minh CityHo Chi Minh City, Vietnam; 11 Shaikh Zayed Postgraduate Medical InstituteLahore, Pakistan

**Keywords:** Asia, insulin, Type 2 diabetes mellitus

## Abstract

**Background:**

The First Basal Insulin Evaluation (FINE) Asia study is a multinational, prospective, observational study of insulin-naïve Type 2 diabetes mellitus (T2DM) patients in Asia, uncontrolled (A1c ≥ 8%) on oral hypoglycemic agents, designed to evaluate the impact of basal insulin initiation.

**Methods:**

Basal insulin was initiated with or without concomitant oral therapy and doses were adjusted individually. All treatment choices, including the decision to initiate insulin, were at the physician's discretion to reflect real-life practice.

**Results:**

Patients (*n*= 2679) from 11 Asian countries were enrolled (mean [±SD] duration of diabetes 9.3 ± 6.5 years; weight 68.1 ± 12.7 kg; A1c 9.8 ± 1.6%). After 6 months of basal insulin (NPH insulin, insulin glargine, or insulin detemir), A1c decreased to 7.7 ± 1.4%; 33.7% patients reached A1c <7%. Fasting blood glucose (FBG) decreased from 11.7 ± 3.6 to 7.2 ± 2.5 mmol/L and 36.8% of patients reached FBG <6.1 mmol/L. The mean daily insulin dose prescribed increased marginally from 0.18 to 0.23 U/kg per day at baseline to 0.22–0.24 U/kg per day at Month 6. Mean changes in body weight and reported rates of hypoglycemia were low over the duration of the study.

**Conclusions:**

Initiation of insulin therapy is still being delayed by approximately 9 years, resulting in many Asian patients developing severe hyperglycemia. Initiating insulin treatment with basal insulin was effective and safe in Asian T2DM patients in a real-world setting, but insulin needs may differ from those in Western countries.

## Introduction

The prevalence of Type 2 diabetes mellitus (T2DM) is increasing worldwide, but the rate of increase is particularly rapid in Asian countries. In the US, the prevalence of diagnosed diabetes increased from 2 to 6% in the 40-year period from 1960 to 2000, approximating a 1% increase per decade.^1^ In contrast, the prevalence of T2DM in China has tripled from 3.2% in 1996^2^ to recent estimates of 9.7% in 2010.^3^ This pattern is repeated across the Asian continent with three- to fivefold increases in the prevalence of T2DM over the past 30 years reported in India, Indonesia, Korea, and Thailand.^4^

In addition to a rapid rate of increase, the diabetes epidemic in Asia is characterized by a relatively young onset and low body mass index (BMI). Asian individuals show a higher percentage of body fat and greater abdominal obesity compared with Western patients with an equivalent BMI.^5^,^6^ The tendency of Asian patients to develop T2DM at a younger age, and so suffer longer with diabetes-associated complications than Western patients,^5^ makes the need for effective management strategies all the more important in order to minimize the burden of diabetes-associated morbidity and mortality.

Tight glycemic control has been established as the cornerstone of effective diabetes management in European and US studies,^7^–^11^ as well as in studies of Asian patients.^12^ Based on such data, the International Diabetes Federation (IDF) Western Pacific Region has proposed a preferred target of A1c ≤ 6.5% for Asian patients with T2DM.^13^,^14^ The American Diabetes Association (ADA)/European Assocaiton for the Study of Diabetes (EASD) consensus statement^15^ suggests an A1c target of <7% and recommends early initiation of insulin in patients not meeting A1c targets. Indeed, it recommends that basal insulin could be added as soon as possible after the “failure” of diet and exercise plus metformin (i.e. A1c ≥ 7.0% for 2–3 months).^15^ A recent randomized controlled trial has demonstrated that prompt addition of basal insulin to patients with an A1c level of 7–8% on maximal doses of metformin and sulfonylurea provides clinically relevant improvements in glycemic control compared with intensification of lifestyle factors.^16^

However, despite the proven benefits of insulin therapy and existing guidelines for the initiation of insulin therapy, evidence suggests that insulin utilization and glycemic control remain suboptimal. In the Hong Kong Diabetes Registry, of 7549 Chinese patients with T2DM, mean A1c was 7.7 ± 1.8% and most patients (60.3%) had A1c >7.0% despite the fact that many were receiving multiple oral hypoglycemic agents (OHAs).^17^ Moreover, the proportion of patients with inadequate glycemic control on OHAs (A1c ≥ 7%) in that study increased with an increasing duration of T2DM, from 23.7% of patients with diabetes for <5 years to 75.9% of patients a disease duration of ≥20 years.^17^ Similar patterns of inadequate therapy intensification were reported in the DiabCare^18^,^19^ study and the International Diabetes Mellitus Practice Study (IDMPS) registry.^20^ These studies revealed that insulin utilization in Asia has not changed markedly over the past 10 years, despite evolving treatment guidelines advocating the initiation and intensification of therapy to reach A1c goals of <6.5%.^13^ The objective of the First Basal Insulin Evaluation (FINE) Asia study was to provide details on the real-world initial insulinization of patients with T2DM across Asia and to determine the tolerability and efficacy of basal insulin regimens in this region.

## Methods

### Objectives of the registry

The primary objective of the registry was to collect real-world information on the initiation of basal insulin in insulin-naïve T2DM patients inadequately controlled on OHAs in Asia.

### Registry design

Patients in the present multinational, prospective, observational study were enrolled from 195 centers/sites across 11 different Asian countries (Bangladesh, China, Hong Kong, India, Indonesia, Korea, Pakistan, Singapore, Taiwan, Thailand, and Vietnam) from 2006 to 2008.

### Patients

Patients were aged 20 years or older, had inadequately controlled T2DM (A1c ≥ 8%) on OHAs, and required the initiation of a basal insulin based on the judgment of their treating physician.^15^ Patients were ineligible for inclusion in the study if they had been prescribed insulin before the registry period (except for acute rescue insulin therapy) or had been prescribed premixed insulin at the start of the registry period. Women who were either pregnant or of childbearing age and not using a reliable contraceptive for the duration of the study were also excluded from the registry.

### Study treatment and assessments

Basal insulin was initiated with or without concomitant OHAs. No specific protocol was recommended as to the type of basal insulin or OHAs administered, which were prescribed at the discretion of the treating physician. Basal insulin doses were adjusted individually based on the recommendations of the locally approved package inserts. The treatment duration was 6 months.

Efficacy and safety data were collected at baseline and at 3 and 6 months. The registry involved three main visits, namely at inclusion and at Months 3 (±7 days) and 6 (±7 days), in addition to standard clinical visits as deemed appropriate by the patient and physician. Each visit included standard physical examinations (including body weight and blood pressure), assessment of A1c, fasting blood glucose (FBG) and self-monitoring blood glucose (SMBG) profiles, adverse drug reactions (ADRs), and hypoglycemic episodes. Patients were recommended to perform SMBG using their own glucose monitors as per their usual practice. In addition, SMBG was recommended when mild-to-moderate hypoglycemic events occurred. Physician and patient assessments of treatment satisfaction were collected at study end based on a four-point scale of satisfaction rated as “not good”, “moderate”, “good”, and “very good”.

### Primary and secondary endpoints

The primary efficacy endpoint was the change in A1c from baseline to Months 3 and 6 after insulin initiation. Secondary and other efficacy endpoints included the change in FBG from baseline to Months 3 and 6 after insulin initiation, response rates (the percentage of patients reaching A1c <7% or achieving the treatment target), mean insulin doses, number of severe hypoglycemic events, and treatment satisfaction.

Safety was evaluated using the ADRs reported during the registry, including all non-serious ADRs (especially hypoglycemic events), serious ADRs, overdoses, and changes in clinical and/or laboratory data. Severe hypoglycemia was defined as blood glucose (BG) <3.9 mmol/L and requiring assistance. Mild to moderate hypoglycemia was defined as episodes that were suggestive of hypoglycemia with no need for external assistance, with BG <3.9 mmol/L but asymptomatic, or symptomatic hypoglycemia with or without a blood glucose measurement.

### Statistical analysis

Statistical analyses were based on patients with A1c data at both baseline and 6 months. All data were entered into a single database by double data entry and were validated in terms of limits, coding, missing data, and consistency checks. Any missing or incomplete data were queried unless specified as unknown. All data were analyzed in an exploratory manner using sas Version 8.2 (SAS Institute, Cary, NC, USA). Summary statistics (mean, median, range, and SD for continuous variables, and the number and percentage for categorical variables) were determined. Student's paired *t*-test was used to compare parameters before and after the treatment period. Qualitative variables were compared using Fisher's exact probability test or Chi-squared tests. All statistical tests were performed using two-tailed tests at a 5% level of significance or with adjustment if appropriate. A least squares multivariate procedure was used to adjust outcome variables with significant baseline differences across treatment groups.

## Results

### Patient characteristics

**Figure 1:**
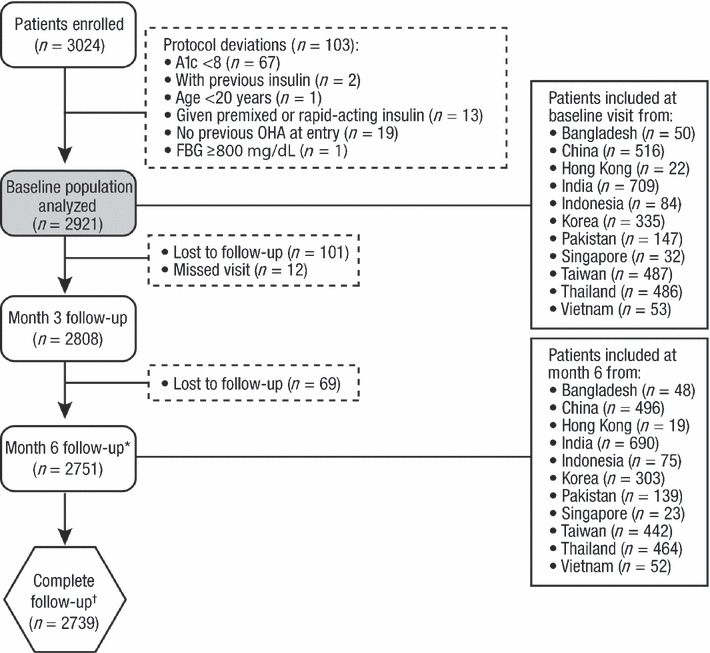
Patient distribution. *Includes patients that missed the visit at Month 3. ^†^Patients with follow-up visits at Months 3 and 6. OHA, oral hypoglycemic agent; FBG, fasting blood glucose.

Across 11 Asian countries, a total of 3024 patients were screened, of whom 103 were deemed ineligible for the present study (Fig. 1). A total of 2921 patients (1452 men and 1469 women, with a mean [±SD] age of 56.4 ± 11.2 years, T2DM duration of 9.3 ± 6.5 years, duration of OHA therapy of 8.7 ± 6.4 years, A1c levels of 9.8 ± 1.6%, and FBG of 11.7 ± 3.6 mmol/L) were included at baseline. Overall, 2808 and 2751 patients attended visits at Months 3 and 6, respectively. Reasons for discontinuation are shown in Fig. 1. One hundred and thirty-one patients treated with insulin glargine (baseline–Month 3, *n*= 82; Month 3–6, *n*= 49), 33 treated with NPH insulin (*n*= 15 and 18, respectively), and six treated with insulin detemir (*n*= 4 and 2, respectively) were lost to follow-up.

The baseline characteristics and clinical characteristics of the total evaluable population (patients who had baseline and Month 6 A1c data; *n*= 2679) are given in Tables 1 and 2, respectively. At the screening visit, 2196 patients (75.2%; evaluable *n*= 2016) were prescribed insulin glargine, 637 (21.8%; evaluable *n*= 589) were prescribed NPH insulin, and 75 (2.6%; evaluable *n*= 61) were prescribed insulin detemir. Thirteen patients (0.5%; evaluable *n*= 13) started another type of insulin. The study population represents a broad range of patients with T2DM and the general characteristics were, on the whole, comparable between the countries involved. However, there were some differences in patient characteristics among groups of patients according to insulin prescribed, including diabetes duration, duration of OHA treatment, and FBG levels (Table 2), but age and BMI were comparable. As indicated in Table 3, 80.8% of patients (*n*= 2360) were using combination OHA therapy before the baseline visit; this percentage decreased at the time of insulin initiation (*n*= 2012; 68.9%). The mean (±SD) doses of insulin at baseline were 0.20 ± 0.09 U/kg for insulin glargine, 0.18 ± 0.11 U/kg for NPH insulin and 0.23 ± 0.10 U/kg for insulin detemir; the type and doses of insulin remained stable at Months 3 and 6 (Table 3).

**Table 1:** Patient characteristics at baseline

Parameter	Glargine (*n*= 2016)	NPH insulin (*n*= 589)	Insulin detemir (*n*= 61)	Total (*n*= 2679)
Gender				
No. men (%)	1041 (51.6)	258 (43.8)	28 (45.9)	1334 (49.8)
No. women (%)	975 (48.4)	331 (56.2)	33 (54.1)	1345 (50.2)
Age (years)	56.6 ± 11.2	55.5 ± 11.2	56.1 ± 9.7	56.4 ± 11.2
Weight (kg)	69.0 ± 12.6*	65.1 ± 12.0	67.9 ± 13.6	68.1 ± 12.7
Mean BMI (kg/m^2^)	26.3 ± 4.9*	25.3 ± 4.1	26.0 ± 4.6	26.1 ± 4.7
BMI				
>23 kg/m^2^	1513 (77.2)	414 (70.8)	46 (75.4)	1983 (75.7)
>25 kg/m^2^	1104 (56.4)	289 (49.4)	37 (60.7)	1435 (54.8)

Data are presented as the mean ± SD or the number of subjects in each group with percentages given in parentheses, as appropriate. Note, the percentages were calculated based on the number of patients with evaluable data rather than the total eligible population.

* *P*< 0.0001 compared with NPH insulin.

BMI, body mass index.

**Table 2:** Clinical characteristics at baseline

	Glargine (*n*= 2016)	NPH insulin (*n*= 589)	Insulin detemir (*n*= 61)	Total (*n*= 2679)
Duration of diabetes (years)	9.5 ± 6.5*†	8.2 ± 6.0	10.9 ± 7.2	9.3 ± 6.5
Duration of OHA treatment (years)	8.9 ± 6.3*†	7.7 ± 5.9†	10.6 ± 7.1	8.7 ± 6.3
A1c (%)	9.7 ± 1.5*	10.1 ± 1.9	10.2 ± 1.7	9.8 ± 1.6
FBG (mmol/L)	11.6 ± 3.5	12.0 ± 3.6	12.6 ± 3.2	11.7 ± 3.5
SBP (mmHg)	134.1 ± 17.1*	132.5 ± 17.3	132.4 ± 16.5	133.8 ± 17.2
DBP (mmHg)	81.3 ± 10.5*†	79.2 ± 10.1	77.0 ± 10.3	80.8 ± 10.4
Diagnosed hypertension	1342 (66.9)	358 (61.0)	44 (72.1)	1754 (65.8)
Coronary artery disease	293 (15.5)	64 (11.4)	11 (18.3)	370 (14.6)
Stroke	84 (4.4)	29 (5.0)	1 (1.7)	114 (4.4)

Data are presented as the mean ± SD or the number of subjects in each group with percentages given in parentheses, as appropriate. Note, the percentages were calculated based on the number of patients with evaluable data rather than the total eligible population.

* *P*< 0.0001 compared with NPH insulin

† *P*< 0.0001 compared with insulin detemir.

OHA, oral hypoglycemic agent; FBG, fasting blood glucose; SBP, systolic blood pressure; DBP, diastolic blood pressure.

**Table 3:** Changes in oral antihyperglycemic agents and insulin during the study

			Current therapy	
			
	Prior therapy	Initial visit	Month 3	Month 6
None	0 (0)	50 (1.7)	N/A	N/A
Monotherapy	561 (19.2)	859 (29.4)	N/A	N/A
Combination*	2360 (80.8)	2012 (68.9)	N/A	N/A
2	1503 (51.5)	1488 (50.9)	N/A	N/A
3	765 (26.2)	484 (16.6)	N/A	N/A
4	85 (2.9)	40 (1.4)	N/A	N/A
5	7 (0.2)	0 (0)	N/A	N/A
Type				
Sulfonylureas	2507 (85.8)	2135 (73.1)	1982 (70.6)	1848 (67.2)
Biguanides	2259 (77.3)	1979 (67.8)	1846 (65.7)	1724 (62.7)
Thiazolidinediones	777 (26.6)	639 (21.9)	574 (20.4)	558 (20.3)
α-Glucosidase inhibitors	501 (17.2)	474 (16.2)	434 (15.5)	443 (16.1)
Meglitinides	170 (5.8)	216 (7.4)	219 (7.8)	258 (9.4)
Others	24 (0.8)	4 (0.1)	4 (0.1)	10 (0.4)
None	0 (0)	50 (1.7)	80 (2.9)	83 (3.0)
Insulin glargine	0 (0)	2196 (75.2)	2038 (72.6)	1952 (71.0)
NPH insulin	0 (0)	637 (21.8)	587 (20.9)	553 (20.10)
Insulin detemir	0 (0)	75 (2.6)	68 (2.4)	63 (2.3)
Other insulin	0 (0)	13 (0.5)	62 (2.2)	101 (3.7)
No insulin			53 (1.9)	82 (3.0)
Mean (±SD) insulin dose (U/kg)				
Insulin glargine		0.20 ± 0.09	0.21 ± 0.10	0.22 ± 0.10
NPH insulin		0.18 ± 0.11	0.21 ± 0.13	0.23 ± 0.14
Insulin detemir		0.23 ± 0.10	0.23 ± 0.09	0.24 ± 0.10

Data are presented as the mean ± SD or the number of subjects in each group with percentages given in parentheses, as appropriate.

* Numbers refer to the number of drugs (listed below in “Type”) used in combination by patients.

### Glycemic control

**Figure 2:**
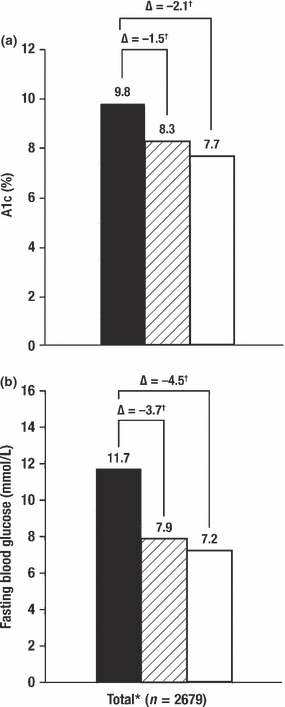
(a) A1c and (b) fasting blood glucose levels at baseline (▪) and Months 3 (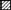) and 6 (□) adjusted for baseline. *Includes 13 patients treated with insulin listed as starting another type of insulin. Data are mean values. ^†^*P*< 0.0001 compared with baseline. Δ, change from baseline.

In the total study population at Month 6, A1c was significantly lower than at baseline (*P*< 0.0001; Fig. 2a). At similar daily insulin doses, baseline-adjusted A1c levels were reduced by 2.2% with insulin glargine, 1.9% with NPH insulin, and 0.8% with insulin detemir, whereas the unadjusted A1c reductions were 2.1%, 2.1%, and 1.1%, respectively. The overall proportion of patients who achieved an A1c target <7% at Month 6 was 33.7% (35.2% with insulin glargine, 30.7% with NPH insulin, and 9.8% with insulin detemir). Similarly, the mean FBG level in the total population was significantly lower at Month 6 compared with baseline (all *P*< 0.0001; Fig. 2b), as were those with insulin glargine, NPH insulin, and insulin detemir. Unadjusted reductions in FBG for insulin glargine, NPH insulin, and insulin detemir were 4.5, 4.5, and 3.6 mmol/L, respectively. An FBG target of <6.1 mmol/L at Month 6 was achieved by 36.8% of total patients (39.5% with insulin glargine, 29.7% with NPH insulin, and 23.0% with insulin detemir).

### Body weight

Overall, the change in body weight was minimal (mean change −0.06 kg). For patients receiving insulin glargine, NPH insulin, and insulin detemir, baseline-adjusted changes in body weight were −0.04, −0.13, and −0.01 kg, respectively; unadjusted changes were −0.02, −0.21, and −0.01 kg, respectively. Changes in body weight in those who achieved A1c <7% (*n*= 901) were not significant, with a mean change in weight of 0.06 ± 2.68 kg for a mean change in A1c of 2.85 ± 1.40%.

### Hypoglycemia and ADRs

A total of 389 patients (14.5%) reported ADRs, including 233 (11.6%) with insulin glargine, 141 (24.9%) with NPH insulin, 12 (19.7%) with insulin detemir, and three (23.1%) with other insulins. Most events were episodes of hypoglycemia, with only five ADRs other than hypoglycemia reported. The incidence of any hypoglycemic event in the total study population over 6 months of treatment was 0.287 events/patient-year (mild-to-moderate 0.280 events/patient-year; severe 0.009 events/patient-year). According to insulin treatment received, the incidence (events/patient-year) of mild-to-moderate and severe hypoglycemia at Month 6 were 0.224 and 0.003, respectively, for those on insulin glargine; 0.458 and 0.031, respectively, for those on NPH insulin; and 0.361 and 0.000, respectively, for those on insulin detemir.

Of the five ADRs other than hypoglycemia reported, all were mild in severity. Among patients receiving insulin glargine, one experienced injection site numbness, which resolved after changing injection site, and a second patient had low-grade fever that subsided on discontinuation. In those receiving NPH insulin, one patient experienced edema, which disappeared on discontinuation, and a second patient had an injection site skin reaction, also resulting in discontinuation. One patient receiving insulin detemir reported dull pain, which recovered following a change in dose.

### Treatment satisfaction

Treatment satisfaction was high for both physicians and patients. A total of 2661 responses from a maximum total of 2679 (response rate 99.3%) were recorded, with most physicians rating insulin treatment as either good or very good (34.2% and 37.4%, respectively). Across the different insulins, the proportion of physicians reporting “good” or “very good” was 33.9% and 42.5%, respectively, for insulin glargine; 36.9% and 21.4%, respectively, for NPH insulin; and 22.9% and 19.7%, respectively, for insulin detemir.

A total of 2658 patients responded to the satisfaction question from a maximum total of 2679 (response rate 99.2%). The overall percentage of patients that rated insulin treatment as either “good” or “very good” was 41.3% and 35.6%, respectively. For individual treatments, the corresponding figures were 40.0% and 41.3% for insulin glargine; 47.6% and 20.7% for NPH insulin; and 27.9% and 23.0% for insulin detemir.

## Discussion

The results of the present prospective, observational, registry-based study showed that initiation of basal insulin in Asian patients with long-standing T2DM failing OHA therapy provided clinically important improvements in glycemic control. However, the present study also reveals that despite the clinical benefits and the recommendations of international treatment guidelines,^13^–^15^ the initiation of insulin therapy in Asia is still being delayed for too long, resulting in many patients developing severe hyperglycemia.

Our study population comprised patients with poorly controlled T2DM, with a mean A1c level of 9.8%, a mean duration of diabetes of 9.3 ± 6.5 years, and a mean duration of OHA therapy of 8.7 ± 6.3 years before insulinization. Approximately one-third of patients had an A1c level >10%. This confirms that treatment intensification and the initiation of insulin therapy is still being delayed in Asian patients, irrespective of international and regional guidelines.^13^,^14^ This finding is not unique to Asian countries. The Cardiovascular Risk Evaluation in People with Type 2 Diabetes on Insulin Therapy (CREDIT) registry of 3031 patients who recently started insulin therapy in Northern America, Europe, and Asia found that the mean duration of T2DM was 11 years and the mean A1c level at baseline was 9.5%.^21^

Initiation of basal insulin in the present study resulted in a statistically and clinically significant reduction in mean A1c levels from 9.8 to 7.7% over 6 months. As a result, one-third of patients reached the ADA/EASD consensus A1c target of <7%. The mean basal insulin dose of 0.22–0.24 U/kg required to achieve this 2% reduction in A1c was low compared with studies in Western populations. For example, in the Treat-to-Target study of North American patients with T2DM, the mean change in A1c was approximately −1.6% over 6 months with insulin doses of 0.48 U/kg (47.2 IU) for insulin glargine and 0.42 U/kg (41.8 IU) for NPH insulin.^22^ Meanwhile, in a study of European patients, the mean change in A1c was −0.96% with bedtime insulin glargine compared with −0.84% with NPH insulin over 6 months, with insulin doses reaching 39 and 37 IU at endpoint, respectively.^23^ The lower insulin doses in the present study may reflect the generally lower BMI of Asian compared with Western T2DM patients. The mean BMI in the present study was 26.1 kg/m^2^, compared with approximately 28 kg/m^2^ in the European study^23^ and 32 kg/m^2^ in the Treat-to Target study of North American patients.^22^ This observation is consistent with a large population-based study of 3071 Asians and 129 116 non-Hispanic Whites in the US.^6^

Thus, Asian patients with T2DM may have lower insulin needs compared with non-Asian populations. Nevertheless, only one-third of patients treated with basal insulin glargine in the present study reached an A1c <7.0%. Patterns of insulin titration observed in the present study may be reflective of a cautious approach to insulin titration among physicians in Asia. Trials of insulin in Asian subjects report using a conservative titration goal relative to comparative trials in Western populations owing to a perceived increased risk of hypoglycemia in Asian patients^24^ who are leaner than their Western counterparts. Rates of hypoglycemia in the present study were low and it is tempting to speculate that more aggressive dose titration, as recommended by the IDF Western Pacific Region,^13^,^14^ may have resulted in more patients achieving the <7.0% A1c target.

It should be noted that observational registry-based studies, such as the present study, have a number of advantages and disadvantages compared with randomized controlled trials. The major advantages of a registry are the potential for larger-scale trials with greater numbers of patients and the ability to monitor therapy under “real-life” conditions that may better reflect how the treatment is used in practice. However, registry studies are not randomized and the characteristics of patients receiving the different treatment modalities may not be consistent; there may also be some differences between the use of insulins within each country or by each physician. Thus, conclusions about the comparative efficacy of NPH insulin, insulin glargine, and insulin detemir in an Asian population cannot be drawn from the present observational study.

Indeed, there were marked differences in the number of patients who were prescribed each of the insulins in the present study (*n*= 2196, 637, and 75 for insulin glargine, NPH insulin, and insulin detemir, respectively). This may reflect the availability or awareness of the different insulins among some of the countries included here; for example, insulin detemir was not available in China and Vietnam at the time of the study and was a new addition to clinical practice in many of the other countries. Physicians are less likely to be familiar with the use of insulin detemir than that of NPH insulin and insulin glargine. In particular, insulin detemir has the option for once- or twice-daily dosing and, because some patients require twice-daily treatment to achieve optimal benefit, there may have been a greater risk of suboptimal titration with this agent.

Diabetes duration also varied between patients receiving the different insulins and was longest in those receiving insulin detemir, which may have influenced efficacy outcomes. Variations in baseline A1c levels in patients receiving the different insulins may also have affected the efficacy outcomes, and this is reflected in the differences between unadjusted and adjusted A1c and FBG changes reported in the present study. In general, patients with higher initial A1c levels are known to respond more readily to insulin than those with initial A1c levels closer to target.

It should also be noted that a threshold of A1c ≥ 8% was selected for the present registry study to include only patients who would be candidates for insulin therapy, whereas people with an A1c of 7–8% are more likely to achieve their therapeutic goal with current therapy as an initial step, before adding a new treatment such as insulin.^15^ Accordingly, the characteristics of the patients included in the present study, being an incomplete cross-section of patients with T2DM, do not fully reflect the heterogeneity of the patient population. Nevertheless, we were able to include a large number of patients with unacceptably high A1c levels who should be considered as candidates for treatment modification and intensification.^15^

In addition, the FINE Asia study analysis did not account for variations in the methods used to measure A1c and relied on accurate physician reporting of A1c levels across the countries involved. Furthermore, initiation of the appropriate insulin dose was determined by country-specific prescribing information, which may also have led to procedural variation across the countries in question. Although these are potential limitations to the study analysis, they are reflective of “real-world” clinical practice, which was a key aim of the present study.

It is important to consider the results of the present study in light of the limitations described above. Yet, the data presented here support the findings from prior registry studies^13^,^14^ that insulin therapy is underused and glycemic control is suboptimal in a large proportion of patients with T2DM in Asia. The FINE Asia study investigated basal insulin initiation, which represents only one of several options for patients with T2DM suboptimally controlled with oral agents. Basal insulin therapies studied here were selected at the discretion of the treating physician alone. Thus, our survey confirms, in a real-world setting, the findings from randomized controlled trials that the initiation of basal insulin is an effective and well-tolerated treatment option in Asian patients with T2DM failing to meet targets with OHA therapy. The study also indicates that Asian patients may have lower insulin needs, possibly related to a lower mean BMI than comparable Western populations. However, more aggressive dose titration may enable more patients to achieve treatment targets and so limit the burden of diabetes associated complications among Asian populations.
